# Apocynin, a Low Molecular Oral Treatment for Neurodegenerative Disease

**DOI:** 10.1155/2014/298020

**Published:** 2014-07-22

**Authors:** Bert A. ‘t Hart, Sjef Copray, Ingrid Philippens

**Affiliations:** ^1^Department of Immunobiology, Biomedical Primate Research Centre, Lange Kleiweg 161, 2288 GJ Rijswijk, The Netherlands; ^2^Department of Neuroscience, University Medical Center Groningen, University of Groningen, Antonius Deusinglaan 1, 9713 AV Groningen, The Netherlands

## Abstract

Accumulating evidence suggests that inflammatory mediators secreted by activated resident or infiltrated innate immune cells have a significant impact on the pathogenesis of neurodegenerative diseases. This may imply that patients affected by a neurodegenerative disease may benefit from treatment with selective inhibitors of innate immune activity. Here we review the therapeutic potential of apocynin, an essentially nontoxic phenolic compound isolated from the medicinal plant *Jatropha multifida*. Apocynin is a selective inhibitor of the phagocyte NADPH oxidase Nox2 that can be applied orally and is remarkably effective at low dose.

## 1. Introduction

Ageing societies are facing an increasing prevalence of neurodegenerative diseases. Some relatively prevalent examples are Alzheimer's and Parkinson's disease and less prevalent are Huntington's and Lou Gehrig's disease (amyotrophic lateral sclerosis; ALS). All neurodegenerative diseases have in common that no effective treatment exists that can stop the progressive deterioration of neurological functions. Of note, none of the neuroprotective agents that have been tested in the clinic have an efficacy that goes beyond symptoms control.

In recent years the insight has been growing that inflammatory reactions from resident or infiltrated innate immune cells may have a significant impact on the pathogenesis of neurodegenerative disorders [1]. A recognized central player is the microglia, a glial cell that belongs to the myeloid lineage and is often indicated as the macrophage of the CNS. This intriguing new insight may imply that drugs with proven efficacy in the protection of peripheral organs against the cytotoxic function of innate immune cells, such as mononuclear or polymorphonuclear phagocytes (resp., MNCs and PMNs), might also be useful for the treatment of the neuroinflammatory component of neurodegenerative diseases. A major hurdle that drugs need to take is to cross the blood brain barrier and penetrate the CNS parenchyma where the neurodegenerative process takes place. In this review we will discuss preclinical studies highlighting the potential of apocynin, a small phenolic antioxidant, as treatment of neurodegenerative diseases.

## 2. Apocynin, a Pharmacologically Active Plant Phenol

Apocyin (4′-hydroxy-3′-methoxyacetophenone or acetovanillone) was identified as the biologically active substance in the roots of* Picrorhiza kurroa* Royle ex Benth, a perennial plant growing in the alpine Himalaya. Extracts from the roots are used in the Ayurvedic medical tradition of India and Sri Lanka for the preparation of ethnic medicines for the treatment of ailments of liver, heart, joints, and lungs. We have prepared a 95% ethanolic root extract under controlled conditions in the laboratory and subjected the preparation to an activity-guided purification using the oxidative burst of human polymorphonuclear/neutrophilic granulocytes (PMN) as an experimental test for acute inflammation [2].

**Figure 1:**
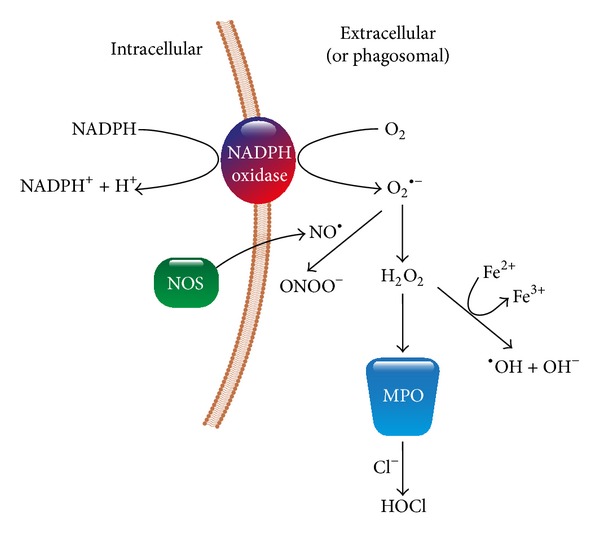
Reactive oxygen species produced in the phagocyte oxidative burst. MPO: myeloperoxidase; NOS: nitric oxide (NO) synthase.

The read-out assay we used was based on the generation of luminol-enhanced chemiluminescence by human PMN stimulated with zymosan opsonized in human serum. The essence of the assay is that the serum-opsonized yeast particles stimulate the PMN via surface-exposed receptors of immunoglobulins or complement factors. The activation signals relayed via these receptors lead to the emptying of cytoplasmic granules (degranulation) and the assembly of the phagocyte NADPH oxidase Nox2. The Nox2 enzyme complex is assembled from membrane-bound (gp91^phox^, p22^phox^) and cytoplasmic (p40^phox^, p47^phox^, p67^phox^, and Rac2) subunits [3]. The assembly process involves phosphorylation of subunits by specific kinases and formation of thiol-bridges. The assembled complex takes up electrons from NADPH and transfers these onto free molecular oxygen leading to formation of superoxide anion (O_2_^−^; one electron reduction) and hydrogen peroxide (H_2_O_2_; two electron reduction). Both oxidants have cytotoxic activity as could be shown using red blood cells from different species [4]. The oxidative burst of PMN comprises a cascade of strongly reactive oxygen species, collectively indicated as ROS (Figure 1). By reaction of O_2_^−^ with nitric oxide the strongly cytotoxic peroxynitrite is formed. In the presence of Fe^2+^ ions H_2_O_2_ is converted into highly reactive hydroxyl radicals (OH^•^), which via peroxidation of membrane lipids affect the fluidity of cell membranes. Myeloperoxidase released by degranulation of the PMN catalyzes the reaction of H_2_O_2_ with halide molecules (Cl_2_, Br_2_, and J_2_) forming highly toxic hypohalides (OCl^−^, OBr^−^, and OJ^−^). ROS are essential components of the intracellular killing of phagocytosed microbes, but when released into the extracellular milieu they are important mediators of the tissue destructive activity of activated PMNs [5, 6].

**Figure 2:**
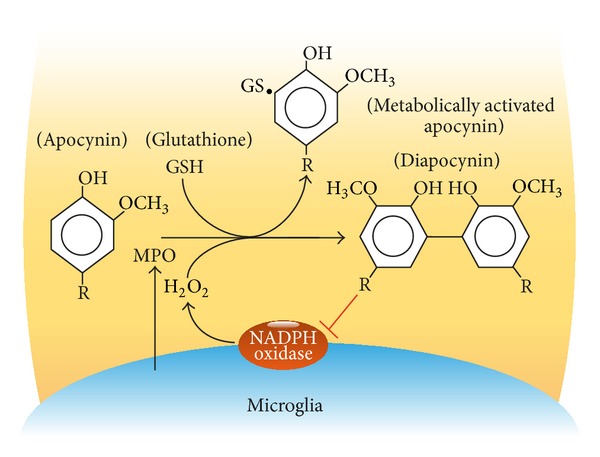
*Inhibition of microglia Nox2 by metabolically activated apocynin*. Receptor-mediated activation of microglia cells induces production of reactive oxygen species and release of myeloperoxidase (MPO). The MPO-catalyzed reaction of apocynin with H_2_O_2_ leads to production of a reactive intermediate that stabilizes by binding to free thiol groups, for example, GSH, or by dimerization. Dimeric apocynin (diapocynin) inhibits Nox2 activity.

It can be envisaged that components of chemically complex plant extracts can interfere with the read-out assay at multiple levels and may also exert nonspecific effects such as killing of the PMN or scavenging of the oxyradicals. This implies that successful activity-guided purification needs to be well focused and carefully controlled for nonspecific effects to avoid false positive results. Notwithstanding these hurdles, we were able to demonstrate a highly specific activity of apocynin in the assay. Apocynin was found to be metabolically activated in an MPO-catalyzed reaction with H_2_O_2_ [7] forming a symmetrical dimer, diapocynin [8] (Figure 2). The observation that the reaction intermediate could be trapped with GSH led us to hypothesize that metabolically activated apocynin might block the formation of thiol bridges between the membrane-bound and cytosolic components that assemble functional Nox2. It was later found, however, that diapocynin directly inhibits Nox2 superoxide production and that this activity is independent of MPO [8]. An important finding with apocynin has been that it inhibits the oxidative burst of PMNs, without impeding the intracellular killing of bacteria. This implies that treatment with apocynin may prevent collateral damage to tissues infiltrated by activated PMN without impeding their bactericidal function.

## 3. Efficacy of Apocynin in AIMID Animal Models

The initial target disease in which we tested the clinical effect of apocynin was the WAG/Rij (RT-1^u^) rat model of collagen-induced arthritis (CIA), which is an accepted preclinical model of the autoimmune inflammatory disease (AIMID) rheumatoid arthritis (RA). In this model, PMNs have a clear pathogenic role [9], reflecting the situation in RA patients [6]. In the rat study we chose to administer apocynin at a dose range of 0,3 to 200 *μ*g/mL drinking water, which was provided ad libitum. It was observed that already at the lowest dose of 0,3 *μ*g/mL, corresponding to a daily oral dose of 6 *μ*g, the arthritis was almost completely suppressed [10]. No effect of apocynin on serum levels of anti-collagen autoantibody or of IL-6, an important pathogenic cytokine in CIA and RA, was observed, suggesting high selectivity for the inflammatory component of the disease. Independent from us, Hougee et al. demonstrated in a mouse CIA model that orally administered apocynin restores the blocked production of cartilage proteoglycan in the arthritic joint [11]. An intriguing side effect of the treatment, illustrating the powerful anti-inflammatory effect of apocynin, was the dramatic suppression of the necrotizing skin lesions at the sites where the immunizing antigen/CFA formulation was injected [12].

Since its initial identification as potent anti-inflammatory agent in 1990, apocynin has become an established inhibitor of the oxidative burst in neutrophils as demonstrated in a wide range of* in vivo* models for immune-mediated inflammatory disorders affecting peripheral and central organs. Of particular importance for this review are the promising clinical effects observed in models of neurodegenerative disease, including ALS, Alzheimer, and Parkinson's disease. In these models the antioxidant activity of apocynin is not targeted to the neutrophil but to the “macrophage of the brain,” that is, microglia.

## 4. Microglia

The brain contains various cell types with the capacity to exert immune functions including astrocytes, microglia cells, and macrophages located in the meninges and perivascular spaces of brain arteries and capillaries [13]. For their immune tasks these cells are equipped with conserved receptors for pathogen-associated or damage-associated molecular patterns, which relay activation signals to the cells for inducing inflammatory effector mechanisms [14].

Microglia are innate immune cells ubiquitously distributed within the central nervous system where they are engaged in tight interactions with neurons, oligodendrocytes, and astrocytes. However, only microglia release MPO after stimulation, being a requisite for the metabolic activation of apocynin.

Microglia in the healthy CNS have a ramified resting phenotype. Opposite to earlier concepts, microglia in the healthy brain are not resting but are highly dynamic cells that carry out homeostatic surveillance of the extracellular environment by the extension and retraction of their protrusions and phagocytosis of tissue debris, which could otherwise cause inflammation [15]. Activated microglia are found in diseased CNS tissue, such as within demyelinated cortical grey matter lesions in the MS brain, surrounding amyloid plaques in Alzheimer brain and in the degenerating substantia nigra in Parkinson's disease [16, 17]. Although the diverse expression profiles of microglia appear to reflect a broad, continuous spectrum of activation states, two activation states at both ends of the spectrum can be recognized corresponding to the M1 and M2 state designated for macrophages [18]. “Classically activated” M1 microglia, for example, induced by LPS or IFN*γ*, have proinflammatory functions which are exerted by the secretion of proinflammatory cytokines such as TNF-*α*, IL-1*β*, and IL-12 and toxic substances such as reactive oxygen and nitrogen species. “Alternatively activated” M2 microglia, such as induced in a milieu containing high IL-4 or IL-13 levels, have anti-inflammatory and tissue regenerative activities, which are mediated by cytokines such as IL-4, IL-10, or TGF-*β* and repair factors such as insulin-like growth factor, arginase-1, or chitinase-like-1. Not only the cytokine milieu, but also the redox state of the microenvironment, which is directly related to NADPH oxidase activity, determines the functional differentiation of microglia towards an M1 or M2 phenotype [18].

## 5. Apocynin as a Potential Treatment of Neurodegenerative Disease

M1 microglia cells are the main resource of Nox2 in the brain. The expression by M1 microglia of activated Nox2 producing ROS is an essential component of microglia-mediated neurotoxicity. The broadly accepted notion that microglia-derived ROS are important mediators of neurodegenerative brain injury raises the question whether the favorable pharmacological profile and low toxicity of apocynin can be used for neuroprotective treatment. Microglia cells not only express Nox2 but also secrete MPO after activation and could thus potentially exert metabolic activation of apocynin. Several authors have reported on the beneficial effect of apocynin on (models of) acute neurological disorders, such as ischemia, intracerebral hemorrhage, and stroke (reviewed in [19]). What are the perspectives for apocynin in chronic neurodegenerative disorders?

*In vitro* studies using cultured microglia have implicated Nox2-derived ROS in the proliferation and functional polarization of microglia [20, 21]. A crucial finding has been that inhibition of Nox2 promotes alternative and anti-inflammatory microglia activation during neuroinflammation [22]. This implies that suppression of Nox2 with apocynin might restore a healthy balance between a proinflammatory M1 and an anti-inflammatory/proregenerative M2 phenotype of microglia. Others have shown that apocynin lowers the production of IL-1*β*, TNF-*α*, and nitric oxide by microglia, thus interrupting a self-perpetuating cycle of detrimental activity. While the exact neurotoxic mechanism of activated microglia in neurodegenerative disease is still uncertain, it is also of considerable interest that the release of the excitotoxin glutamate requires Nox2 activity and that this can be inhibited by apocynin [23]. Taken together, these data suggest a potentially beneficial role of apocynin in neurodegenerative disease. Indeed, promising effects of apocynin have been observed in mouse models of some major neurodegenerative diseases.

*Amyotrophic Lateral Sclerosis *(*ALS, Lou Gehrig Disease*). Amyotrophic lateral sclerosis is a common adult-onset neurodegenerative disease affecting motor neurons. The disease leads to rapidly progressing motoric impairment and death usually within 5 years. While in the majority of patients the cause of the disease is unknown, in a subset of cases the disease has a genetic cause, namely, mutations in the superoxide dismutase-1 (SOD1) gene [24, 25]. The mutation enhances oxidative stress by dysregulated production of superoxide anion due to reduced dismutation to H_2_O_2_. Mutant SOD1 expressing astrocytes are linked to ALS pathology because of their reduced capacity to absorb glutamate [26] and/or by their release of neurotoxic factors [27]. Selective silencing of mutant SOD1 [28] or replacement of mutant by wild-type microglia [29] in the mutant SOD1 mouse model strongly point to a central pathogenic role of microglia. Harraz et al. have used oral apocynin to control progression of neurodegeneration in a SOD1 mutant mouse model and observed promising effects [30]; it was observed that administration of apocynin in the drinking water significantly prolonged survival and delayed the onset of motoric defects. This study shows that orally administered apocynin can build up a sufficiently high concentration within the CNS parenchyma for mitigating neurotoxic levels of ROS production. However, these promising data could not be reproduced in another study using the same mutant mouse strain [31].

*Alzheimer Disease* (*AD*). Alzheimer disease is an ageing-associated progressive neurological disorder leading to irreversible dementia. Neuropathological hallmarks of AD are senile plaques of misfolded and fibrillar amyloid-*β* aggregates and intraneuronal tangles of tau protein within the cerebral cortex [32]. Activated microglia cells were found clustered around senile plaques producing neurotoxic agents like ROS, NO, and TNF-*α*. Activation of microglia Nox2 by oligomeric and/or fibrillar amyloid-*β* [33, 34] and expression of activated Nox2 in Alzheimer brain [35] have been reported.

Lull et al. have tested apocynin at a daily oral dose of 10 mg/kg via the drinking water in a hAPP(751)_SL_ transgenic mouse model of AD [36]. They observed in apocynin-treated mice a significant reduction of plaque size within cortex and hippocampus and a reduction of microglia numbers in the cortex, but not in the hippocampus. However, a behavioral feature of AD observed in this mouse model, that is, performance in the Morris water maze swim test, which tests spatial memory organized in the hippocampus, was not markedly improved by the treatment. The limited clinical effect of apocynin in the model might be due to the absence of clear neuroinflammation, while this is more prominent in AD patients, and because of the fact that plaque formation does not necessarily predict cognitive decline.

*Parkinson's Disease *(*PD*). The pathological hallmark of PD is a progressive degeneration of dopamine producing neurons in the substantia nigra (SN), a pigmented structure located in the bottom of the midbrain. Via the release of dopamine, the SN has a central role in the coordination of various neurological functions, including reward, addiction, and movement. The latter function is particularly disturbed in PD. To compensate for dopamine loss, a metabolically stable precursor of dopamine (L-DOPA) is given, which in a substantial number of patients causes typical involuntary movements known as hyperkinetic syndrome. While in the vast majority of (sporadic) PD patients the cause of the disease is not known, in a small fraction a genetic cause has been found, namely, mutations in several genes, including alpha-synuclein, parkin, leucine-rich repeat kinase 2, PTEN-induced putative kinase 1, and ATP13A2 [25]. The observation that users of heroin contaminated with MPTP developed PD symptoms [37] enabled generation of a clinically relevant animal PD model. After conversion of MPTP into MPP+ by monoamine oxidase B in astrocytes, MPP+ is concentrated in dopaminergic cells via uptake through the specific dopamine transporter, where it blocks complex I of the mitochondrial respiratory chain. The ensuing redox stress causes amongst others dysregulation of cellular Ca^2+^ leading to cell death. Just like in ALS and AD, neurodegeneration in PD is found to be associated with microglia Nox2 activation, which is thought to contribute significantly to the pathogenic process [38].

Using an* in vitro* system, Gao et al. demonstrated that ROS generated by microglia Nox2 enhances the sensitivity of dopaminergic neurons to MPP+ [39]. A beneficial effect of apocynin on neurotoxic effects mediated by microglia has been shown in a mouse model of PD [40].

## 6. The Effect of Apocynin in a Nonhuman Primate Parkinson's Disease Model

Repetitive injection of a low dose of MPTP in common marmosets, a small-bodied neotropical primate, elicits a neurological disease that at the level of clinical and neuropathological presentation closely approximates PD [41]. We have used this MPTP model in 5 marmoset twins to test whether oral apocynin is also effective in a higher species [35]. For oral administration, apocynin was dissolved in Arabic gum; one sibling of each twin was given apocynin containing gum and the other was given only the gum. Treatment with apocynin (100 mg/kg, TID) started one week before PD induction with MPTP (1 mg/kg, via subcutaneous injection for 8 days). Apocynin limited the typical body weight loss associated with the parkinsonian syndrome. Also the motor function in the apocynin treated monkeys was improved, indicating an anti-Parkinson efficacy of apocynin. Moreover, the number of surviving dopamine neurons was increased by apocynin, indicating a neuroprotective efficacy. Remarkably, apocynin has a similar molecular structure as homovanillic acid (HVA), a metabolite of dopamine. An explanation of the protective efficacy of apocynin in PD might also be related to the compensation of the reduced level of the natural available o-methoxycatechol HVA.

## 7. Perspective for Treatment of Human Patients

Apocynin is a potentially attractive oral prodrug because of its low general toxicity and the fact that its specific antioxidant action is elicited after metabolic activation by MPO releasing phagocytic cells. Safety data of apocynin are scarce, but those available show low toxicity and high stability (partly reviewed in [19]). The LD50 after oral dosing in mice has been estimated at 9 g/kg. In rats about 80% of intraperitoneally injected apocynin at 120 mg/kg was recovered in unchanged form in a urine sample collected 20 hours later. An intravenous dose of 420 mg/kg apocynin in mice caused minimal signs of toxicity [12].

To our knowledge, apocynin has not been tested in human neurodegenerative disease patients. However, Peters et al. have evaluated the therapeutic potential of inhaled apocynin on ozone-induced bronchial hyperresponsiveness to methacholine in asthmatic patients as a model of inflammatory lung disease [42]. The authors could exclude scavenging of ozone by apocynin and concluded that the effect was mitigating ROS production by PMNs and eosinophils that had infiltrated the lung upon ozone exposure.

The mouse studies discussed in this review show that low doses of apocynin administered via the oral route reach the CNS parenchyma in a sufficient concentration to inhibit the microglia oxidative burst and inhibit neurodegeneration. Taking the very low systemic toxicity and the highly specific mode of action of apocynin into account it would be an attractive perspective to test the therapeutic value in human neurodegenerative disease.
